# Challenges in Diagnosing Extruded Hydrogel Scleral Buckle Mimicking an Orbital Abscess: A Case Report

**DOI:** 10.7759/cureus.56371

**Published:** 2024-03-18

**Authors:** John K Jung, Anne C Chan, Rodney Guiseppi, Brian Wong

**Affiliations:** 1 Department of Ophthalmology, University of Texas Medical Branch at Galveston, Galveston, USA

**Keywords:** retinal detachment repair, scleral buckle extrusion, scleral buckle complication, retinal surgery, hydrogel scleral buckle

## Abstract

In this case report, we present an unusual complication linked to a hydrogel explant in a 72-year-old male presenting to the emergency department with persistent left eye pain, redness, and discharge for one month. The patient had a history of retinal detachment in 1989, which was managed with scleral buckle surgery and gas injection. Initial examination revealed an extruding scleral buckle in the superior temporal region, along with signs of an infection. CT scans revealed a 1.9 × 1.2 × 3.8 cm abscess accompanied by preseptal cellulitis. This case report highlights the importance of how hydrogel scleral buckle explants may mimic the presentation and symptoms of an abscess as a long-term complication. Nevertheless, there have been several reports of long-term issues associated with the expansion of the hydrophilic hydrogel material. This case report further illustrates how complications linked to hydrogel explants can resemble abscess symptoms, underscoring the significance of accurate diagnosis and appropriate management.

## Introduction

Scleral buckle surgery has been one of the main methods for rhegmatogenous retinal detachment (RRD) for over 60 years to seal the retinal break(s) and assist retinal reattachment [1]. Since proven to be effective, various materials have been utilized for scleral buckling, including fascia lata, palmaris tendon, plantaris tendon, polyethylene, gelatin, hydrogel, and silicone [2]. In the 1980s, an episcleral hydrogel explant was introduced as a new alternative to silicone explants for treating RRD due to its potential to minimize scleral erosion and prevent postoperative infections through its antibiotic properties [3]. Hydrogel explants were shown to have minimal postoperative complications six to 53 months after surgery; however, long-term complications related to the swelling properties of the implant were reported, such as infection, ptosis, strabismus, and other orbital complications [4,5]. This case study aims to report long-term complications of hydrogel scleral buckle explants and the following diagnostic challenges.

## Case presentation

A 72-year-old male presented to the emergency department with a chief complaint of left eye pain, redness, and discharge for one month. The symptoms started 10 days prior to presentation. The patient noticed the development of a mass in his left eye along with a copious mucopurulent discharge accompanied by blurry vision, which he attributed to this discharge. Significant ocular history included a retinal detachment in 1989 (at 39 years old), which was repaired with a scleral buckle and gas injection. Despite his retina being attached after the surgery, his vision remained suboptimal in that eye. In the following years, a visible swelling was noticed by his ophthalmologist around the site of the scleral buckle, which was monitored given that he was asymptomatic. The patient was then lost to follow-up prior to his presentation to the emergency room. At the time of presentation, his review of systems was unremarkable, and the symptoms were localized to the affected eye.

**Figure 1:**
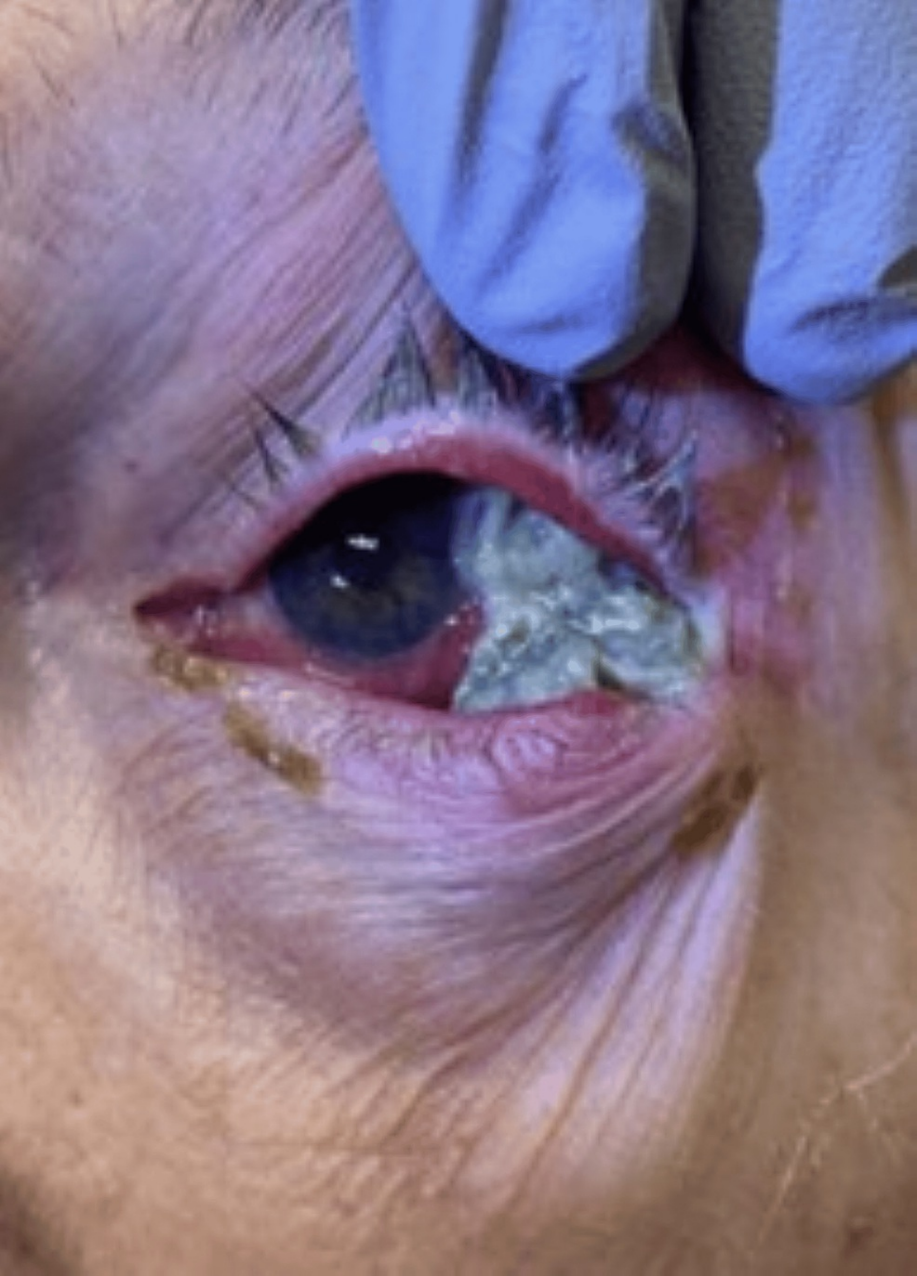
Extruded scleral buckle with overlying mucopurulent discharge in the superior and temporal quadrants associated with preseptal cellulitis

His presenting visual acuity OS was 20/800 with correction and pinhole to 20/400 with an intraocular pressure of 12 mmHg. Diminished ocular motility across all fields was noted with sluggish and minimal pupillary reflexes. Significant examination findings included upper and lower eyelid erythema with ptosis. The extruded scleral buckle was noted in the superior temporal quadrant of the subconjunctival space, with significant mucopurulent discharge associated with conjunctival injection (Figure 1). The cornea was clear, the anterior chamber was deep and quiet, and there were 360 degrees of posterior synechiae with pigments on the anterior lens capsule. The retina was attached to an area of cryo-retinopexy scar in the superior temporal quadrant; no evidence of vitritis or exudative retinal detachment was noted, and the optic nerve was well perfused.

**Figure 2:**
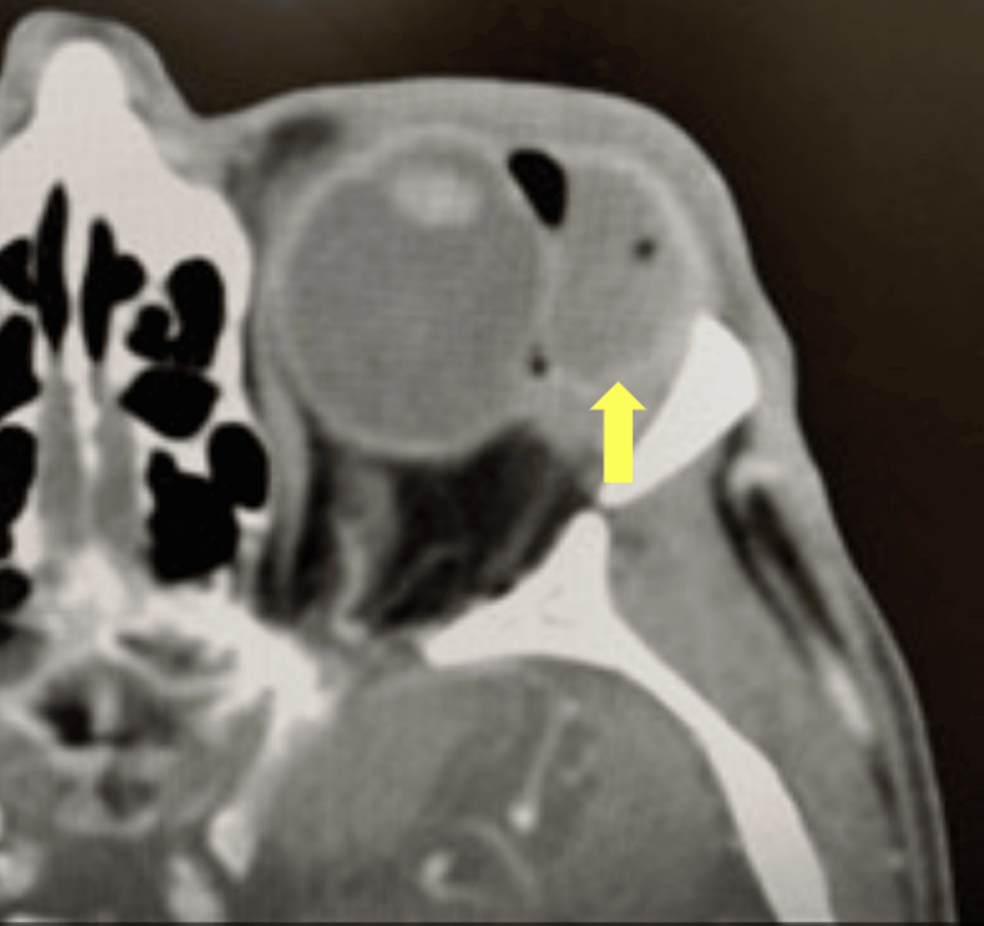
Axial CT scan of the orbit with superior temporal mass mimicking an abscess with a mass effect on the temporal globe The arrow illustrates the superior temporal mass mimicking an abscess in the axial CT scan. The mass was noted to be the swollen hydrogel scleral buckle.

Given the initial suspicion of a superimposed orbital abscess secondary to an infected extruded scleral buckle, a CT of the orbits with and without contrast revealed a 1.9 × 1.2 × 3.8 cm fluid collection in the region of the left lacrimal gland (Figure 2). The fluid collection resulted in compression of the globe, and there was overlying preseptal cellulitis. Given the initial diagnosis of an orbital abscess secondary to an extruded scleral buckle, the patient was started on fortified vancomycin and ceftazidime eye drops with intravenous ampicillin/sulbactam and consented to scleral buckle removal and drainage of the orbital abscess with the vitreoretinal and oculoplastic services.

The patient was taken to the OR for an incision, drainage of the abscess, and removal of the extruded scleral buckle. Intraoperatively, it was noted that the scleral buckle was made of hydrogel and was distended without any evidence of an orbital abscess. The buckle was removed in pieces using blunt instruments, cotton swabs, irrigation, and suction, and the area was inspected thoroughly for any residual foreign bodies and then irrigated with vancomycin and basic salt solution. It was decided to leave the area open due to an active infection. The sclera remained stable and intact, not requiring any patch grafts. The patient did well postoperatively, and his uncorrected vision improved to 20/50, and his retina remained attached. The wound culture grew *Streptococcus anginosus* and *Streptococcus intermedius*, which were both sensitive to penicillin. The patient was discharged on oral amoxicillin-clavulanate in addition to topical prednisolone acetate eye drops and tobramycin-dexamethasone ointment. The patient was, however, lost to follow-up after he was discharged.

## Discussion

Hydrogel explants were introduced as a potential alternative to silicone explants due to their perceived benefits in minimizing scleral erosion and preventing postoperative infections, attributed to their antibiotic properties [3]. However, long-term complications related to the expanding size of the hydrophilic hydrogel material have since been reported in numerous cases.

This case illustrates that hydrogel explant complications can mimic the presentation of orbital cellulitis with an abscess. Orbital cellulitis with an abscess was considered because the hydrophilic nature of the hydrogel scleral buckle allowed the material to swell and increase in volume. The absorption of water in the scleral buckle resulted in the extrusion of the scleral buckle and also caused ocular motility restriction. Secondly, the radiographic features on the CT scan demonstrated a homogenous swelling that looked like a fluid collection mimicking an orbital abscess.

A study in 2007 that quantified the incidence of MIRAgel (hydrogel) buckle explant complications (415 patients) that required scleral buckle removal (27 patients, 6.5%) reported that reasons for removal were redness and discharge (93%); extrusion of the buckle (22%); subconjunctival mass and/or protrusion of the buckle beneath the eyelids (70%); strabismus, rectus muscle palsy, limitation of ocular motility, and/or diplopia (67%); and orbital fullness and/or pseudotumor (22%). After MIRAgel removal, the retina remained attached in 85% of patients with stabilized visual acuity, while retinal redetachment was found in 15% of the 27 patients [6]. Previous literature and studies suggest that long-term complications related to MIRAgel buckle swelling are due to its hydrophilic nature [3-6]. This study highlights the importance of recognizing that patients who had scleral buckle explants done in the 1980s may have hydrogel buckles, and recognizing the complications associated with them will help optimize management.

## Conclusions

In this case study, we underscore the importance of awareness among professionals regarding the potential complications arising from hydrogel scleral buckles, especially in patients with a remote history of scleral buckling and an unusual inflammatory or infectious presentation. The unique nature of hydrogel material, which can expand due to its hydrophilic properties, necessitates a high index of suspicion for complications such as extrusion and mimicking of infections or abscesses. This awareness is pertinent for accurate diagnosis and intervention. Not only does this case add to the growing body of literature documenting long-term complications of hydrogel scleral buckles, but it also serves as a reminder of the importance of a detailed patient history and the need for ongoing surveillance of patients with hydrogel implants.
